# Establishing *Trypanosoma cruzi* farnesyl pyrophosphate synthase as a viable target for biosensor driven fragment‐based lead discovery

**DOI:** 10.1002/pro.3834

**Published:** 2020-02-07

**Authors:** Giulia Opassi, Helena Nordström, Arne Lundin, Valeria Napolitano, Francesca Magari, Tom Dzus, Gerhard Klebe, U. Helena Danielson

**Affiliations:** ^1^ Department of Chemistry—BMC Uppsala University Uppsala Sweden; ^2^ SciLifeLab Uppsala Sweden; ^3^ BioThema Handen Sweden; ^4^ Malopolska Centre of Biotechnology Jagiellonian University Krakow Poland; ^5^ Faculty of Biochemistry, Biophysics and Biotechnology Jagiellonian University, Gronostajowa Krakow Poland; ^6^ Institut für Pharmazeutische Chemie Phillips‐Universität Marburg Marburg Germany

**Keywords:** binding efficiency, Chagas disease, farnesyl pyrophosphate synthase, fragment screening, SPR, Trypanosoma cruzi

## Abstract

Procedures for producing and exploring *Trypanosoma cruzi* farnesyl pyrophosphate synthase (tcFPPS) for surface plasmon resonance (SPR) biosensor‐driven fragment‐based discovery have been established. The method requires functional sensor surfaces with high sensitivity for extended times and appropriate controls. Initial problems with protein stability and lack of useful reference compounds motivated optimization of experimental procedures and conditions. The improved methods enabled the production of pure, folded and dimeric protein, and identified procedures for storage and handling. A new coupled enzymatic assay, using luciferase for detection of pyrophosphate, was developed and used to confirm that the purified enzyme was active after purification and storage. It also confirmed that sensor surfaces prepared with structurally intact protein was active. An SPR‐biosensor assay for fragment library screening and hit confirmation was developed. A thermal shift assay was used in parallel. A library of 90 fragments was efficiently screened by both assays at a single concentration in the presence and absence of the catalytic cofactor Mg^2+^. Hits were selected on the basis of response levels or Δ*T*_*m*_ > 1°C and selectivity for tcFPPS in the presence of Mg^2+^. Characterization of hits by SPR showed that all had low affinities and the relationships between steady‐state responses and concentrations were not sufficiently hyperbolic for determination of K_D_‐values. Instead, ranking could be performed from the slope of the linear relationship at low concentrations. This pilot screen confirms that the procedures developed herein enables SPR‐biosensor driven fragment‐based discovery of leads targeting tcFPPS, despite the lack of a reference compound.

**Significance Statement:**

To enable the discovery of drugs, it is essential to have access to relevant forms of the target protein and valid biochemical methods for studying the protein and effects of compounds that may be evolved into drugs. We have established methods for the discovery of drugs for treatment of American Trypanosomiasis (Chagas disease), using farnesyl pyrophosphate synthase from *Trypanosoma cruzi* as a target.

**Abbreviations**
AMP: Adenosine monophosphate
CD: circular dichroism
DSF: differential scanning fluorimetry
EDTA: Ethylenediaminetetraacetic acid
FPPS: farnesyl pyrophosphate synthase
FBDL: fragment‐based lead discovery
GPP: geranyl pyrophosphate
IPP: isoprenyl pyrophosphate
nDSF: intrinsic differential scanning fluorimetry
SDS‐PAGE: sodium dodecyl sulfate polyacrylamide electrophoreses
SPR: surface plasmon resonance
tc.: *Trypanosoma cruzi*
TCEP: tris(2‐carboxyethyl)phosphine

## 1 INTRODUCTION

American Trypanosomiasis, known also as Chagas disease, is a neglected tropical disease caused by the protozoan parasite *Trypanosoma cruzi*. Current treatments for Chagas disease are around 40 years old and suffer from long regimens and difficulties in assessing their efficacy for patients in a chronic stage of infection.^1^ However, recent studies of the parasite have identified several targets for new drugs. Our focus is on farnesyl pyrophosphate synthase (FPPS, EC 2.5.1.10), a key enzyme in the mevalonate pathway. It catalyzes the condensation of isoprenyl pyrophosphate (IPP) with dimethylallyl pyrophosphate and with geranyl diphosphate (GPP), essential components for the viability also of *Trypanosoma* species.^2^ FPPS has been proposed as a suitable molecular target for drug development.^3^ Nitrogen containing bisphosphonates, such as risendronate, have antiproliferative and cytocidal effects against *T. cruzi*, resulting from blocking isoprenoid synthesis at the level of FPPS.^4^, ^5^, ^6^ Current efforts are mainly focused on the improvement of bisphosphonates in order to increase their specificity for the parasitic enzyme.^7^, ^8^ Our aim is to facilitate a broader approach in the discovery of drugs by developing effective methods that can identify inhibitors with new scaffolds and/or other modes of action, and with a high potential for efficient and safe drugs.

Fragment‐based lead discovery (FBLD) is a relatively fast and cost‐efficient approach for identifying new leads. The rationale is to use sensitive biophysical methods to screen compound libraries of low molecular mass “fragments” (< 300 Da) for candidates that interact with the target, and to subsequently evolve them into drug leads by structure‐guided optimization.^9^, ^10^ Due to the size of fragments, chemical space can be efficiently sampled using libraries containing few (<2000) but structurally diverse compounds. A major challenge is that fragments typically have very weak affinities for the target of interest and screening methods with a very high sensitivity are required.^11^ Success is typically a result of high‐quality preparations of the target protein (not necessarily large quantities) and an extensive understanding of its physicochemical properties under various conditions. Consequently, a range of biophysical and biochemical methods, target variants and tool compounds, are required for validating the quality of the target sample and for exploring various experimental design strategies.

For the discovery of new leads targeting FPPS, we selected a surface plasmon resonance (SPR) biosensor‐based strategy. SPR is one of the most used biophysical techniques in fragment‐based screening and lead optimization. The technology is based on the detection of the binding between a surface‐immobilized molecule (such as a protein) and a molecule in solution (such as a fragment). A major challenge for SPR‐based screening and lead discovery is the immobilization of a fully functional target on a sensor chip and to keep it functionally stable over the time under the conditions required for experiments (typically days and room temperature). This is particularly critical for screening of fragment libraries due to the typically weak affinities of hits. They often interact nonspecifically, that is, at multiple sites of the target proteins, even when the protein is not correctly folded. It is consequently essential that the target protein is of high quality and that experimental conditions for its handling are under control before performing SPR experiments. This requires expertise and experience in protein production and assessment of the structural integrity (e.g., folding and homogeneity) and functionality of the target (e.g., catalytic activity of enzymes), as well as in how to set up high‐quality assays with suitable analysis protocols.^12^ Reference compounds with known interaction features are used to confirm that sensor surfaces are functional and identified hits interact in the expected manner, taking into account how known references interact.

The cloning and overexpression of *Trypanosoma cruzi* farnesyl pyrophosphate synthase (tcFPPS) in bacteria (*E. coli*) was reported for the first time in 2001.^4^ The same study included a purification protocol and activity assay based on radiometric end point measurements.^13^ The expression/purification and storage conditions described in the initial publication have been repeated successfully in other studies,^14^, ^15^, ^16^ although authors have stated that they encountered some stability issues with the crystallization of the protein.^16^ From our own experience, the protein we initially produced using a very similar approach was unstable and lost functionality over time. It was therefore not of sufficient quality for SPR experiments, especially not for fragment screening. Here, we describe modified expression and storage conditions that enabled the production of high levels of high‐quality protein that could tolerate long‐term storage. We also describe orthogonal procedures for evaluation of both the structural integrity and the activity of tcFPPS. A screen of a small fragment library, using SPR biosensor and thermal shift assays, validated that the produced protein met the requirements for SPR‐driven FBLD.

## 2 RESULTS

### 2.1 *Production of recombinant tcFPPS*

tcFPPS with an N‐terminal His‐tag was recombinantly produced in *Escherichia coli* and purified by Ni^2+^‐IMAC. The purified protein was >90% pure, as estimated by sodium dodecyl sulfate polyacrylamide electrophoresis (SDS‐PAGE) analysis. The final concentration was 7 mg/mL in a total volume of 4.5 mL, giving a total yield of around 30 mg/L of culture.

### 2.2 *Thermal stability of recombinant tcFPPS under different conditions*

Experiments with the tcFPPS initially produced, essentially according to a published protocol,^14^ indicated that the purified protein was unstable and lost functionality over time. It was therefore necessary to optimize the buffers used for storage and subsequent experiments. The optimization was guided by the analysis of the thermal stability of the protein under various conditions, using two different methods that monitor the unfolding of proteins as the temperature is raised. The inflection point/transition between folded and unfolded state serves as a quantitative measure of the melting temperature (T_m_). Differential scanning fluorimetry (DSF) is an indirect method as it measures the exposure of hydrophobic regions of the protein via a fluorescent reporter dye, while intrinsic differential scanning fluorimetry (nanoDSF, nDSF) directly monitors the intrinsic fluorescence of the protein at 350 and 330 nm, i.e. changes in the environment of tryptophan and tyrosine residues.

**Figure 1:**
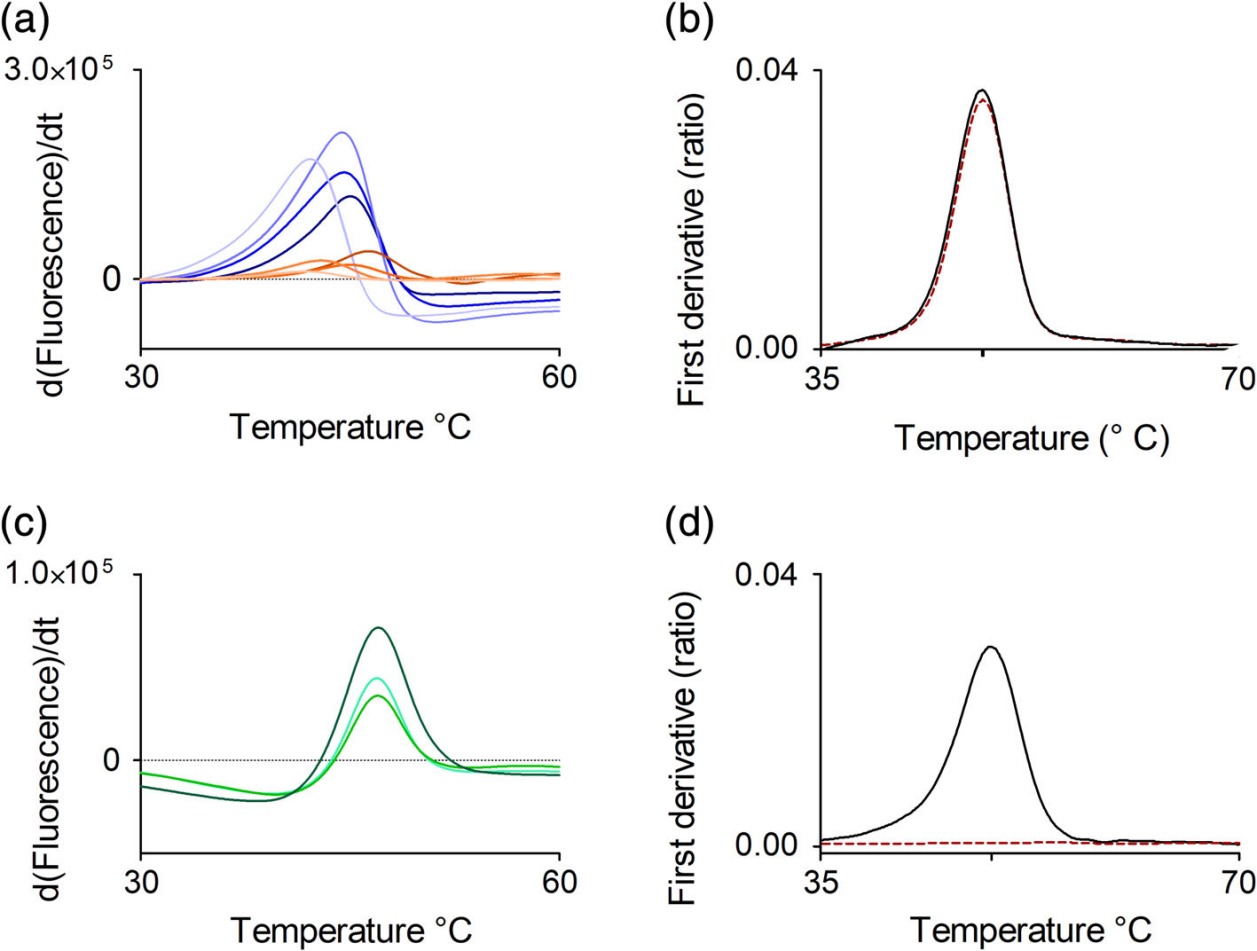
Thermal shift analysis of *Trypanosoma cruzi* farnesyl pyrophosphate synthase (tcFPPS) stability in different buffers. (a) Differential scanning fluorimetry (DSF) analysis of effect of storage buffer composition: 25 mM TRIS (blue) and 25 mM HEPES (orange) at pH 6.5, 7.0, 7.5, and 8.0 (from dark to light shade). (b) nDSF analysis of stability of tcFPPS stored in 25 mM TRIS–HCl buffer at pH 6.5 and 75 mM NaCl, supplemented with 1 mM TCEP at 4°C. Freshly thawed sample (solid line) and after 1 week from thawing (dashed line). (c) DSF analysis of effect of additives in the running buffer used for surface plasmon resonance analysis (10 mM HEPES, 150 mM NaCl, 3 mM Mg^2+^‐acetate) on the stability of tcFPPS. Inclusion of 1 mM TCEP alone (bottom, medium green), 0.05% Tween20 alone (top, dark green), or both 1 mM TCEP and 0.05% Tween20 (middle, light green). (d) nDSF analysis of stability of tcFPPS in sodium acetate pH 5.0 (used as preconcentration solution for immobilization on sensor surface). Freshly protein sample (solid line) and sample thermally inactivated through incubation at 92°C for 5 min (red dotted line)

The thermal stability of tcFPPS was evaluated by DSF in TRIS and HEPES buffers at pH (6.5, 7.0, 7.5, and 8.0; Figure 1a) and at different salt concentrations (data not shown). The experiments revealed that the enzyme was most stable in the acidic buffer with a relatively high ionic strength (75 mM salt). The protein was subsequently stored in 25 mM TRIS–HCl buffer at pH 6.5 and 75 mM NaCl, supplemented with 1 mM tris(2‐carboxyethyl)phosphine (TCEP). nDSF analysis confirmed that the buffer selected for storage of tcFPPS was suitable also for non‐frozen samples. The *T*_*i*_ of a sample kept at 4°C for 1 week after thawing was only reduced by 0.1°C compared to a freshly thawed sample (48.6 and 48.5°C, respectively, Figure 1b).

DSF was also used to evaluate the effect of additives in the standard buffers used for SPR experiments on the stability of tcFPPS (Figure 1c). The experiments showed that the enzyme was relatively stable under these conditions, with an inflection point for thermal denaturation of the protein at around 44°C. It increased by 2°C when TCEP was included in the standard buffer, whereas it was slightly reduced (Δ*T* = 1°C) when Tween20 was included. Finally, the stability of tcFPPS in sodium acetate pH 5.0, used as preconcentration solution for immobilization on sensor surface was also performed by nDSF analysis (Figure 1d). It confirmed that the protein was structurally intact also at these conditions.

### 2.3 *Evaluation of folding and structural homogeneity of tcFPPS*

**Figure 2:**
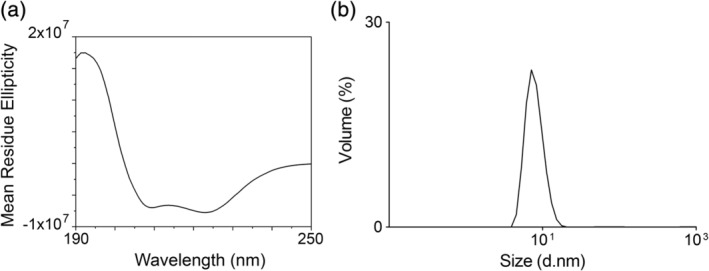
Assessment of folding and structural homogeneity of produced *Trypanosoma cruzi* farnesyl pyrophosphate synthase (tcFPPS). (a) Circular dichroism spectrum of tcFPPS diluted in water to 0.2 mg/mL. (b) Dynamic light scattering analysis of stock solution

To further confirm that the produced tcFPPS had well‐defined regions of secondary structure, as expected from a properly folded protein, circular dichroism (CD) analysis was performed.^17^ The protein sample was diluted in water to avoid high background absorption from buffers at wavelengths below 240 nm. The CD spectrum showed a high peak around 190 nm, while two negative peaks were seen at 210 and 222 nm (Figure 2a). The spectrum was interpreted as a folded structure dominated by alpha‐helices, small beta‐turns and 20% random coil, in accordance with a properly folded protein as indicated by the crystal structure.^16^

The quaternary structure and homogeneity of the purified protein was evaluated by dynamic light scattering (DLS). The technique estimates the molecular weight and size distribution of the protein via measurements of its diffusion in solution over time.^18^ A “distribution by volume analysis” of the protein (stock solution) indicated that there was essentially only a single species of protein present (Figure 2b). The hydrodynamic diameter of the protein was estimated to be 7.14 nm, corresponding to the molecular weight of a dimer (84 KDa).

### 2.4 *Evaluation of tcFPPS activity*

**Figure 3:**
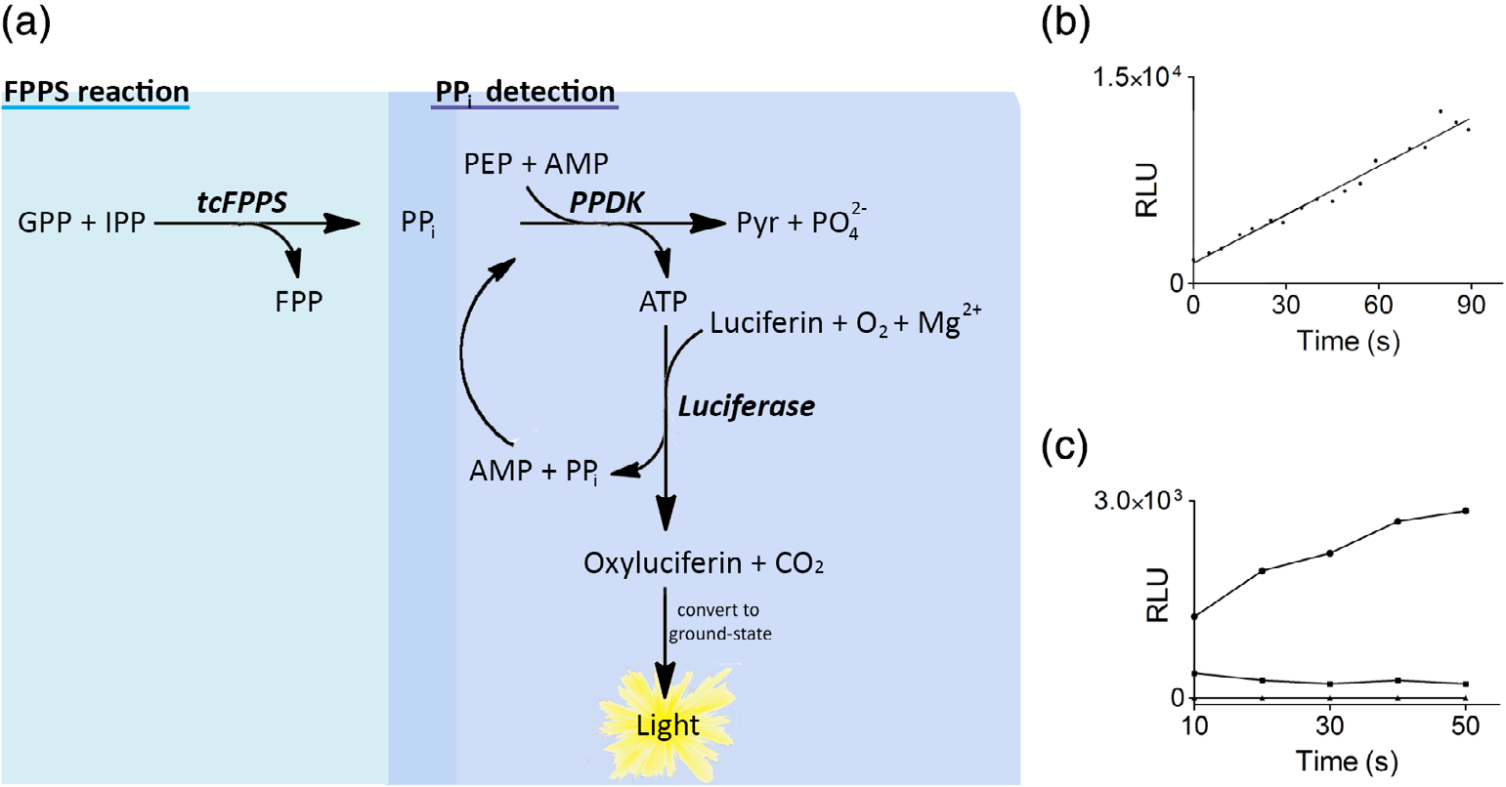
Analysis of catalytic activity of *Trypanosoma cruzi* farnesyl pyrophosphate synthase (tcFPPS). (a) Scheme of coupled enzymatic assay for analysis of tcFPPS activity. tcFPPS reaction: Synthesis of farnesyl pyrophosphate (FPP) and pyrophosphate (PPi) from geranyl pyrophosphate (GPP) and isopentenyl diphosphate (IPP). Pyruvate phosphate dikinase (PPDK) reaction: Conversion of PPi into ATP using AMP, and with the associated conversion of phosphoenol pyruvate (PEP) into pyruvate (Pyr) and phosphate (PO_4_^2−^). Luciferase reaction: ATP drives the conversion of luciferin into oxyluciferin using O_2_ and Mg^2+^. This is followed by the spontaneous reaction that generates light. AMP and pyrophosphate are recycled into the PPDK reaction. (b) Enzymatic activity monitored continuously as the increase in luminescence (relative light unit, RLU) as a function of time (in seconds) in a standard assay mixture consisting of 10 nM tcFPPS, 0.32 U PPDK, luciferase/luciferin reaction kit containing Mg^2+^, 0.5 mM PEP, 1 μM GPP and 2.5 μM IPP, 0.4 mM AMP in HEPES‐EDTA at pH 7.0. (c) Activity of tcFPPS immobilized to a full sensor chip surface on the bench. Reagents were pipetted directly to the chip surface. Samples were extracted every 10 s, and the luminescence was measured according to the standard procedure in microplates. Activities were determined directly after immobilization (top, circles), after a 2‐min incubation with 40 μM risendronate (middle, squares), and after a 2‐min incubation with guanidinium‐HCl (bottom, triangles)

An enzymatic assay for evaluating the catalytic activity of tcFPPS continuously by bioluminescence was developed (Figure 3). The three‐enzyme coupled assay (Figure 3a) monitored the amount of pyrophosphate (PP_i_) released upon the synthesis of FPP, via two serial detection steps that convert formed PP_i_ into a light signal. In the first detection step, the enzyme pyruvate phosphate dikinase (PPDK) uses PP_i_ to form pyruvate and ATP from phosphoenol pyruvate (PEP) and AMP.^19^, ^20^ In the second detection step, luciferase uses the produced ATP and molecular oxygen to convert luciferin into oxyluciferin, with the concomitant release of light. The PP_i_ released also in this step is recycled and used by PPDK to produce ATP, making the assay more stable. The reaction conditions were optimized with respect to the different requirements of the three enzymes involved (tcFPPS, PPDK, and luciferase). All three enzymes were catalytically active in a HEPES buffer at pH 7.0. Although both luciferase and FPPS require Mg^2+^ for activity, it was possible to use an EDTA‐containing reaction buffer optimized for luciferase stability, as both luciferase and FPPS were functional under the reaction conditions used.

The luminescence signal of the reaction mixture increased linearly for over 90 s in the presence of tcFPPS but not in its absence (Figure 3b). The initial rate was proportional to the concentration of tcFPPS and IPP/GPP but was not affected by changes in the concentration of PEP or PPDK (Table 1). This confirmed that the produced enzyme was catalytically active and that the rate‐determining step of the reaction was catalyzed by tcFPPS under these conditions.

**Table 1:** Effect of variations in enzyme and substrate concentrations on catalytic rate of coupled FPPS reaction

	Concentration	Rate (RLU/sec)
tcFPPS (nM)	0	1.8 ± 0.5
	1	8.1 ± 0.1
	5	46 ± 13
	**10**	108 ± 2
IPP/GPP (μM)	0:0	1.2 ± 0.9
	**2.5**:**1**	108 ± 2
	25:25	844 ± 90
PEP (mM)	0.01	106 ± 8
	**0.5**	108 ± 2
	1	117 ± 1
PPDK (U)	**0.32**	108 ± 2
	0.6	113 ± 10

*Note*: tcFPPS was 0, 1, 5, and 10 nM, 1 μM GPP, 2.5 μM IPP, and 25 μM. PEP was 0.01, 0.5, and 1 mM. PPDK was 0.32 and 0.6 U. The standard concentrations are highlighted in bold. Errors are standard deviations, based on triplicate measurements.

Abbreviations: GPP, geranyl pyrophosphate; IPP, isoprenyl pyrophosphate; PEP, phosphoenol pyruvate; PPDK, pyruvate phosphate dikinase; tcFPPS, *Trypanosoma cruzi* farnesyl pyrophosphate synthase.

### 2.5 *Fragment library screening against tcFPPS*

Two methods were used to screen a fragment library against the produced tcFPPS, the first employed an SPR biosensor and the second DSF. These methods are complementary and therefore suitable for orthogonal validation of hits. Ninety structurally diverse fragments with a broad chemical diversity and high solubility were selected for the screening.

**Figure 4:**
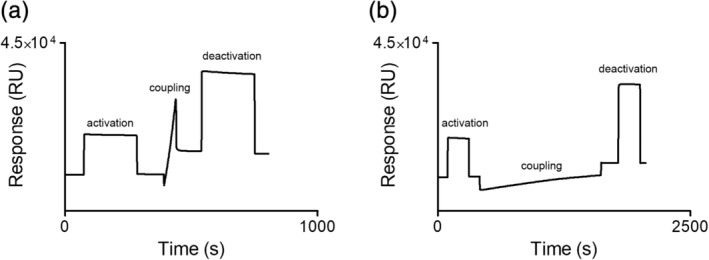
Typical sensorgrams for preparation of a *Trypanosoma cruzi* farnesyl pyrophosphate synthase biosensor surface used for fragment library screening. Sensor surface with active protein (a), and reference surface with thermally inactivated protein (b)

An SPR assay suitable for screening of fragments against tcFPPS was developed by immobilizing the protein by amine coupling (Figure 4a). The level of immobilization was kept to approximately 3,000 RU, theoretically adequate for the detection of interacting fragments. A control immobilization with thermally inactivated FPPS was performed to confirm that the protein needed to be structurally intact for immobilization (Figure 4b). The unfolded protein is not efficiently preconcentrated on the surface, and only low levels were immobilized even after a longer coupling period. Problems with the structural integrity of a protein sample could therefore be detected already at the stage of immobilization.

The screening conditions were based on a standard biosensor buffer, that is, HEPES at pH 7.4, supplemented with 150 mM NaCl and TCEP, carefully selected with respect to enzyme stability/functionality over time (evaluated as described above), physiologically relevant ionic strength and reducing environment. Experiments with three bisphosphonates (risendronate, ibandronate, and alendronate) showed that they interacted with the surface but resulted in secondary effects (not shown). This indicates that the immobilized enzyme was functional, although the compounds were not suitable as reference compounds for screening or characterization of hits.

To confirm that the immobilized enzyme was functional, it was also manually immobilized to the full sensor chip surface on the bench (i.e., outside the instrument), using the same procedure as in the instrument (see above). The catalytic activity was then determined using a modified luminescent assay. The immobilized enzyme was found to be catalytically active, while its activity was significantly lower in the presence of a bisphosphonate inhibitor and lost completely after exposure to guanidinium hydrochloride (Figure 3c).

Two independent SPR screening experiments were performed, using new surfaces prepared specifically for each screen. As there was no suitable reference compound available, the threshold for definition of hits was set to 30% of the theoretical *R*_max_ of each compound. To enable the identification of fragments selective for the Mg^2+^‐bound active form of tcFPPS, the experiments were performed with and without Mg^2+^ in the buffer, with the experiment without Mg^2+^ serving as a negative reference. Fragments interacting with the target in the presence of Mg^2+^ were thus prioritized as hits.

**Figure 5:**
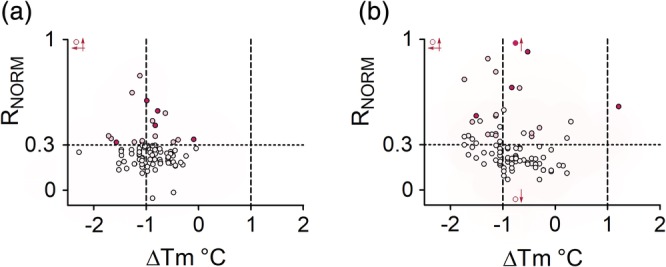
Data from primary screening of fragment library against *Trypanosoma cruzi* farnesyl pyrophosphate synthase using surface plasmon resonance (SPR) and differential scanning fluorimetry, in the (a) presence of Mg^2+^ and (b) absence of Mg^2+^. Data represent normalized SPR signals (R_NORM_ = RU/R_max_). Fragments with signals above the 0.3 R_NORM_ threshold in (a) were defined as hits. Final hits (Compounds 1–5) are shown as dark pink filled circles, 11 additional initial hits are shown as shaded pink circles. These hits have the same coloring in (b)

The SPR‐based screen in the presence of Mg^2+^ initially identified 16 hits, that is, 18% (Figure 5a), of which five (5%) were confirmed by analysis of a concentration series. Hits were considered to be validated if the sensorgrams were of good quality and there was a clear concentration dependence. The five hits were selected although they also interacted with tcFPPS in the absence of Mg^2+^. The observation that 25 fragments were above the threshold in the absence of Mg^2+^ (Figure 5b) suggested that nonspecific binding occurred to a higher degree without Mg^2+^, indicating that Mg^2+^ is important for the structural integrity of FPPS, not only for forming a catalytically competent active site. This interpretation is supported by the much higher signals for the hits in the absence compared to in the presence of Mg^2+^. Consequently, only the five hits confirmed in the replicate screen were considered relevant and were selected for further studies.

Several independent screens were also performed by DSF in the presence and absence of Mg^2+^. The threshold for hit selection was set to Δ*T*_*m*_ > ±1°C, relative the reference *T*_*m*_ obtained by injecting DMSO alone. Most fragments reduced the *T*_*m*_. However, 35 hits were identified both in the presence (Figure 5a) and absence of Mg^2+^ (Figure 5b). Only nine were unique to each condition as 26 fragments were identified under both conditions.

**Figure 6:**
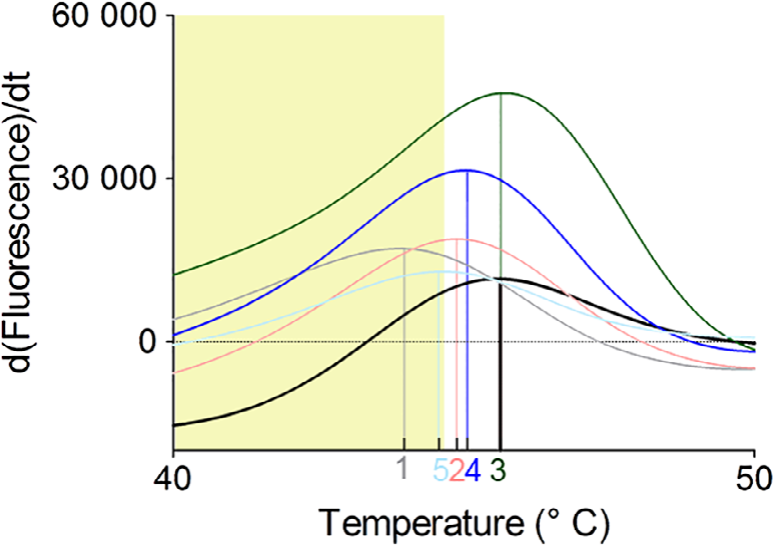
Structural stability of *Trypanosoma cruzi* farnesyl pyrophosphate synthase in the presence of Fragments 1–5 (colored), DMSO reference (black) visualized as differential scanning fluorimetry‐based melting curves. Δ*T*_*m*_ below −1°C of the control is shaded

When comparing the results from the SPR and the DSF screens, it was found that there was a limited overlap between the hits (Figure 5). Of the five hits selected on the basis of SPR experiments, only two resulted in Δ*T*_*m*_ > 1°C when analyzed by DSF in the presence of Mg^2+^ (Fragments 1 and 5 in Figure 6).

**Figure 7:**
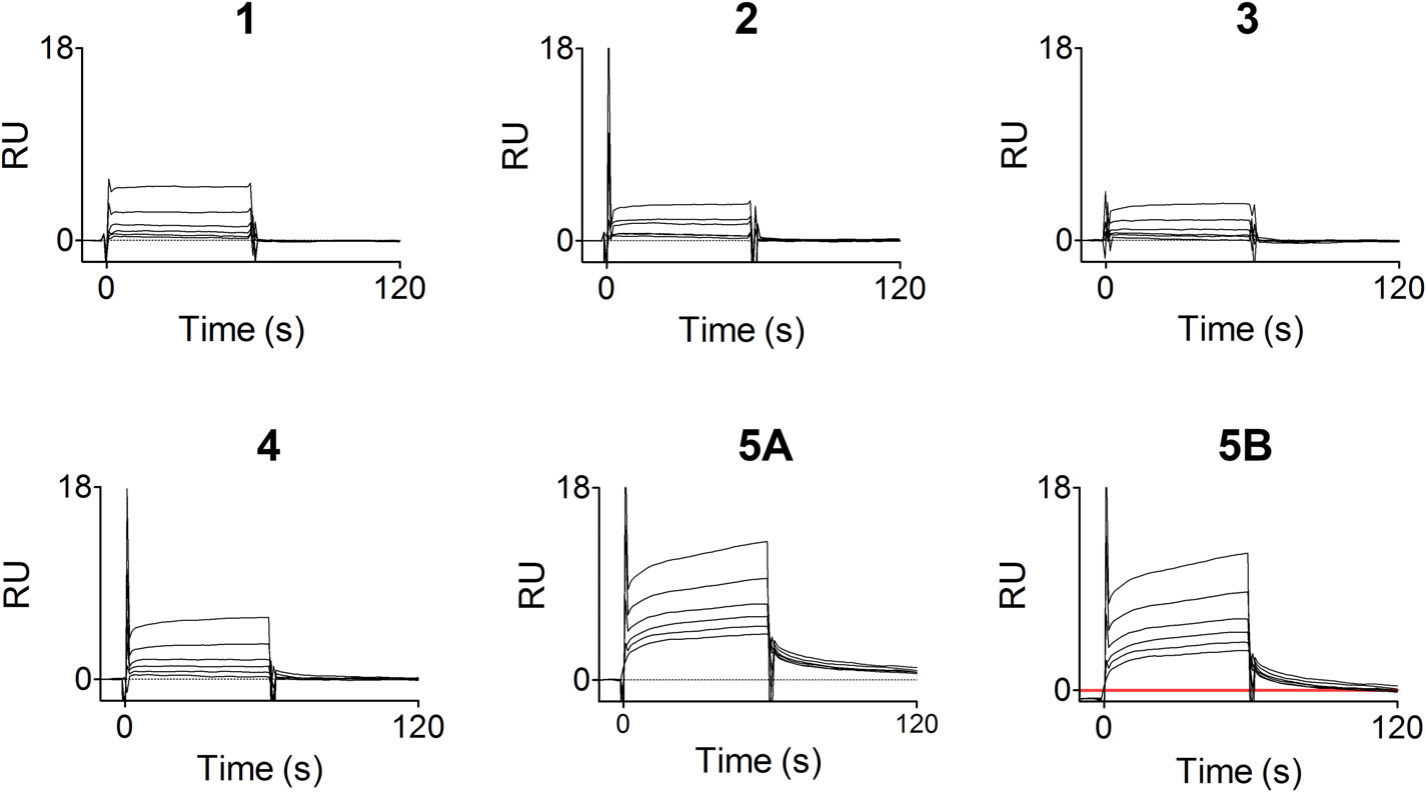
Analysis of interactions between Fragments 1–5 and *Trypanosoma cruzi* farnesyl pyrophosphate synthase, using a surface plasmon resonance biosensor assay. Sensorgrams for threefold dilution series of fragments, starting at 250 μM. For Fragment 5, both the original (5A) and the bulk corrected sensorgrams (5B) are shown

**Figure 8:**
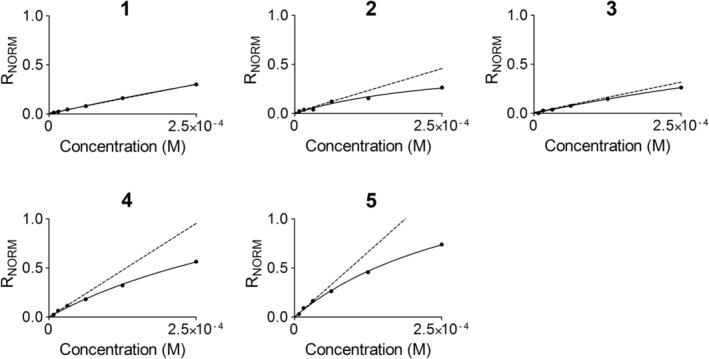
Steady‐state analysis of interactions between Fragments 1–5 and *Trypanosoma cruzi* farnesyl pyrophosphate synthase (data from Figure 7). Solid curves are theoretical saturation curves based on nonlinear regression analysis of reference subtracted and molecular weight normalized (R_NORM_ = RU/Rmax) data and a simple 1:1 interaction. The dashed lines represent the slopes of the graphs at low ligand concentrations, from which the binding efficiency (BE) was estimated. The saturation curve for Fragment 5 was based on bulk corrected sensorgram (5B in Figure 7)

The interaction characteristics of the five selected hits were explored using the SPR biosensor assay. Analysis of a series of concentrations diluted from a maximal concentration of 250 μM resulted in the rapid interaction kinetic profile typical for fragments (Figure 7). Fragment 5 showed secondary effects that could not be eliminated by accounting for a large signal jump at the beginning and end of the injection, potentially due to bulk effects (Figure 5a, b, respectively). The relationship between steady state signals (i.e., representing the complex concentration) and fragment concentrations were analyzed from report points early during the injection, in order to reduce complications from such secondary effects. The relationships were linear or only slightly curved for all fragments, as anticipated for very weak interactions (Figure 8). Although it was technically possible to use the graphs to estimate K_D_ values and calculate corresponding ligand efficiencies (LE) (Table 2), the actual numbers are not relevant since the experiments were performed under conditions far from steady state. *K*_*D*_ values in the mM range represent very weak interactions with the target and extracted signals may be flawed by effects not related to the interaction of interest. In order to rank the fragments on their ability to interact with the target at low concentration, before secondary effects may dominate the signal, the binding efficiency (BE) was therefore also calculated.^21^ The analysis indicated that Fragment 5 binds most efficiently with the target, followed by Fragments 4 and 2.

**Table 2:** Interaction parameters for selected hits

Fragment	*K*_*D*_ (mM)	LE (kcal/mol × HA)	BE (R_NORM_/*M*)
1	0.9	0.3	1.2
2	0.2	0.3	2.0
3	0.3	0.2	1.2
4	0.3	0.4	3.7
5	0.2	0.3	5.3

*Note*: *K*_D_ and binding efficiency (BE) values were estimated from the data visualized in Figure 8. Ligand efficiency (LE) was calculated from *K*_D_ values.

### 2.6 *Inhibition analysis*

The activity of FPPS was analyzed in the presence of the two best hits (Fragments 4 and 5) at 250 μM. However, no effect was detected. In a control experiment, risendronate inhibited the enzyme completely when tested at 10 μM.

## 3 DISCUSSION

To enable the detection of fragments interacting weakly with tcFPPS using biophysical methods, the fundamental first step for FBLD, it was essential to optimize the experimental procedures for producing high‐quality protein and conditions that keep it stable and functional over time. A panel of orthogonal methods was consequently applied for analysis of the produced tcFPPS.

### 3.1 *Production and handling of tcFPPS and quality of produced protein*

Functional tcFPPS was successfully produced and conditions suitable for storage and handling for functional studies and screening were identified. Problems with the initially produced enzyme were attributed to suboptimal conditions for storage and handling. The combination of biophysical methods used confirmed that the revised protocols resulted in the production of an enzyme that was soluble (nonaggregated), in the form of a dimer (84 kDa), folded with secondary structure features in accordance with the crystal structure, and stable over time.^16^

The conditions selected for functional analysis were selected also with respect to physiological relevance. Some of the initial biophysical experiments were performed using buffers suboptimal for functional studies but provided information concerning structural characteristics required at the time. Most important, all buffers used for functional studies contained the catalytic cofactor Mg^2+^ and had a pH of 7.0 or 7.4, close to the cytosolic pH reported for *Trypanosoma Cruzi*, that is, 7.33 for epimastigotes and 7.35 for trypomastigotes.^22^

### 3.2 *Catalytic assay*

The functionality of the enzyme was confirmed by a novel continuous catalytic assay. The conditions for the coupled assay were optimized with respect to the three enzymes involved. EDTA was included in the buffer and the pH was set to 7.0 although this was suboptimal for tcFPPS, being dependent on Mg^2+^ and being maximally active at pH 8.^4^ However, these conditions were considered to be optimal for the luciferase^23^ and PPDK activities.^24^ The resulting assay was found to be dependent on the concentration of tcFPPS, confirming that it represented the rate limiting step of the coupled reaction.

The sensitivity of the developed assay is high, as demonstrated by the possibility of detecting activity with as little as 1 nM enzyme (not shown). It is faster than the radiometric assay currently in use^4^, ^13^ and very practical as it is homogeneous and measurements can be performed in microtiter plates without requiring separate steps for incubation, washing, and so on. The assay is useful both for confirming that the enzyme employed for screening is functional and for discriminating binders from inhibitors, which can be difficult when screening fragments using binding assays. Although risendronate confirmed that the assay can be used to detect inhibition, the identified hits did not show any effect when tested at 250 μM, close to their estimated *K*_*D*_ values. Testing at higher concentrations was not attempted since they would potentially cause side effects by interactions with other components in the assay mixture.

The thermal stability of tcFPPS was sensitive to pH, with a slightly higher stability at pH 6.5 than 7.0 and 7.5. It is marginally below the pH indicated for physiological conditions (of 7.3–7.35).^22^ Furthermore, it has been previously demonstrated that the catalytic activity of the enzyme increases with increased pH, with a maximum at pH 8.0.^4^ Although the pH‐optimum of activity and stability of enzymes are often correlated, there are cases which would suggest just the opposite.^25^ It can be speculated that there is an advantage, maybe from a regulatory perspective, that the pH optimum for stability is different from the optimum for activity.

### 3.3 *Fragment library screening*

The suitability of the produced enzyme for screening of a fragment library using an SPR‐biosensor based assay was validated. The hit rate of 5% is acceptable, and the compounds are of interest to follow up using conventional fragment‐based evolution strategies.

In addition, the results highlight the importance of using high‐quality protein and conditions ensuring that the target is functional in the screening experiment. Often this is monitored by using reference compounds that define the degree of functionality of the sensor surface. But in the absence of suitable reference compounds this is not possible. The functionality of the immobilized protein was therefore evaluated by other means. As a first test, it was confirmed that the low pH conditions (5.0) required for immobilization did not destroy the structural integrity of the protein. The immobilization procedure revealed problems with the structural integrity of a protein sample, and surfaces perceived to be of poor quality were therefore not used. Moreover, immobilization of the protein did not destroy its activity. It was shown to be catalytically active and sensitive to inhibition by bisphosphonates and denaturation by guanidium hydrochloride.

The observation that a larger number of compounds bound in the absence of Mg^2+^ indicated that the degree of nonspecific binding is higher when the cofactor is not present, consistent with its structural effects. It was therefore possible to use the screen performed in the absence of Mg^2+^ as a negative control, but where the identified binders were not selected unless they also bound to the functional surface, instead of selecting compounds that only bound the functional surface, as was the original plan. The hits therefore appear to be specific for the functional form of the protein.

The differences in the results from SPR and DSF can be attributed to the very different principles for selecting hits. DSF only detects hits that interact in regions of the protein which are involved in the folding/unfolding process. The low affinity of fragments also influences the potential to detect binding. The hits from the screen in the presence of Mg^2+^ all exhibited negative Δ*T*_*m*_, while in the screen in the absence of Mg^2+^ hits with positive Δ*T*_*m*_ were detected. This supports that the structural integrity of the protein under the two conditions are very different, influencing the results also with DSF. Artifacts with both false‐positive and false‐negative are well documented for this technique when applied to fragment screening.^11^

Due to the high uncertainty in quantifying low affinity interactions, for which saturation is not reached within a concentration range where the compound is soluble, or when the influence of interactions with secondary sites makes interpretations difficult, the BE was also calculated as a parameter to rank hit compounds.^21^ It is a simple procedure where the initial slope of the curve describing the relationship between the concentrations of complex at different ligand concentrations (Figure 8) is used as a measure of the ligand to bind to the protein without assuming a mechanistic model or stoichiometry. Complexities due to multiple weak interactions (nonspecific binding) or other effects therefore do not come into play. This is an advantage at this stage of hit selection.

### 3.4 *Conclusions*

This first study of FPPS as a target for FBLD has revealed that it can be used for fragment library screening and hit validation using an unconventional referencing, suitable when reference compounds are not available.

## 4 MATERIALS AND METHODS

### 4.1 *Fragment library*

Ninety fragments were selected from a 96‐membered library designed to have compounds with a large chemical diversity and high solubility, suitable for crystallographic driven lead discovery (Frag Xtal Screen, JENA Bioscience) were used.^26^ The screening hits were (Jena compound number, CAS ID): Fragment 1: J3, CAS 838813‐44‐8; Fragment 2: J16, CAS 1311649‐76‐9; Fragment 3: J42, CAS 501442‐73‐5; Fragment 4: J66, CAS 95‐25‐0; Fragment 5: J92, CAS 7659‐29‐2.

### 4.2 *tcFPPS expression and purification*

A plasmid containing the gene for N‐terminally His‐tagged tcFPPS was obtained from Novartis. It was used to transform 100 μl of *E. coli* BL21 (DE3) cells subsequently plated on agarose‐LB solid medium supplemented with kanamycin. Bacterial clones were grown in 2XTY medium containing 50 μg/mL kanamycin at 37°C, until OD_**600**_ of 0.6. The system was cooled to 18°C and growth continued until OD_600_ 1.0, when 1 mM of isopropyl β‐D‐thiogalactoside was added. The cells were harvested by centrifugation after an additional 16 hr of growth at 18°C and frozen at −80°C. After thawing, the pellet was resuspended in phosphate‐buffered saline (PBS; Medicago AB, Uppsala, Sweden) pH 7.4 supplemented with protease inhibitors (cOmplete™ EDTA‐free, Roche), 10 μg/mL DNAse (bovine pancreas, Grade II, Sigma‐Aldrich), 500 μg/mL lysozyme (chicken egg white, Grade VI, Sigma‐Aldrich), 4 mM MgCl_**2**_ (Sigma‐Aldrich), and 5 mM imidazole (Sigma‐Aldrich). The volume of the supplemented PBS buffer was 10 times more than the volume of the pellet itself. Cells were then lysate using a French press (1.7 kPSI) and centrifuged at 18000 rpm for 45 min (4°C).

The supernatant was filtered through a 0.22 μm Millipore filter (Ahlstrom Munksjö, Helsinki, Finland) and loaded onto a Nickel‐chelated agarose affinity column (Qiagen) that had been equilibrated with the same PBS buffer with 5 mM imidazole. Elution of the His‐tagged protein was performed using an increasing concentration of imidazole, as described by the manufacturer protocols.^27^ After concentration via centrifugation using a filter with a cut‐off 30 kDa (Amicon Ultra‐15), the protein was exchanged to a buffer containing 25 mM Tris, 75 mM NaCl, and 1 mM TCEP pH 6.5 using PD10 columns (GE Healthcare). Aliquots of the enzyme were then flash‐frozen in liquid nitrogen and stored at −80°C.

The purity of the protein was estimated by SDS‐PAGE and the concentration by NanoDrop ND‐1000 Spectrophotometer (Marshall Scientific).

### 4.3 *Thermal shift assays*

The thermal stability of tcFPPS was evaluated under different conditions using both indirect and direct thermal shift assays:

**DSF**. An indirect thermal shift analysis was performed using SYPRO Orange and a QuantumStudio 3 instrument (ThermoFisher Scientific). Two different set ups were used:

The stability of tcFPPS was analyzed by adding protein (0.3 mg/mL) to specified buffers, supplemented with SYPRO Orange dye, to a total volume of 20 μL/well. Two different buffering agents (TRIS and HEPES) were tested at 25 mM and different pH values (6.5, 7.0, 7.5, and 8.0) and salt (NaCl from 25 to 200 mM, MgCl_2_ from 0 to 5 mM) concentrations. A thermal ramp of 0.05°C/s was applied from an initial temperature of 20°C to a final temperature of 95°C, the initial and the final temperatures were maintained for 1 min. The system was then cooled down to 20°C at a speed of 1.6°C/s. This setup was applied for identifying suitable storage conditions for tcFPPS and for verifying the stability of the protein in the running buffer chosen for SPR‐based experiments.

A fragment library was screened against 0.21 mg/mL tcFPPS in the screening buffer (10 mM HEPES pH 7.4, 1 mM TCEP, supplemented with 3 mM MgCl_2_ and 1X conc fluorescent dye) in a total volume of 20 μL/well. Each fragment was added to a final concentration 500 μM (2% DMSO). Control experiments with 1 and 2% DMSO were also performed. A thermal ramp of 1°C/min was applied from a starting temperature of 20–95°C, both held for 1 min, as described by Niesen et al.^28^

**nDSF**: Intrinsic protein fluorescence was monitored over a range of temperatures using a Tycho NT6 instrument (Nanotemper Technologies). Two capillaries were filled with identical samples of 7 mg/mL tcFPPS in storage buffer (25 mM Tris pH 6.5, 75 mM NaCl, 1 mM TCEP), directly after thawing from −80°C storage. Duplicate samples that had been kept at 4°C for a week after thawing were analyzed in the same way. An experiment where the stability of tcFPPS in sodium acetate pH 5.0 (used as preconcentration solution for immobilization on sensor surface, see below) was carried out in the same way.

The fluorescence was monitored at 330 and 350 nm during a thermal ramp from 35 to 95°C. The data were plotted as a derivative in order to get the inflection point for the shift of intrinsic fluorescence in the experiments. It was used to calculate the inflection temperature (*T*_*i*_).

### 4.4 *Circular dichroism*

The tcFPPS stock solution was taken from −80°C storage and thawed directly before analysis. It was diluted to a final concentration of 0.2 mg/mL in water. Spectra were recorded from 190 to 250 nm in a 0.2 mm cuvette, using a JASCO 1.500 instrument and analyzed with Spectra Analysis software. Online software DichroWeb^29^, ^30^ was used for the deconvolution of the spectra with Contin‐LL analysis program (Provencher & Glockner method).^31^

### 4.5 *Dynamic light scattering)*

A stock sample of tcFPPS was taken from −80°C storage and thawed directly before analysis. A capillary was filled with the solution of tcFPPS and DLS analysis was performed using Zetasizer Ultra (Malvern Panalytical). The data were analyzed using the Zs Xplorer software (Malvern Panalytical), enabling the estimation of the hydrodynamic radii of the protein using the Stokes‐Einstein equation and the particle size distribution based on the volume of the particles in each size range.^32^

### 4.6 *Catalytic assay*

The activity measurements were carried out in 96‐well microplates (Greiner bio‐one) at 25°C. The standard assay mixture consisted of 10 nM tcFPPS, 0.32 U PPDK (Kikkoman, Osaka, Japan), 40 μL of a luciferase/luciferin assay kit containing luciferase, D‐luciferin, stabilizers and Mg^2+^ (ARSL 11‐501‐TP, BioThema, Handen, Sweden), 1 μM GPP and 2.5 μM IPP (Echelon Inc., Salt Lake City, Utah), 0.5 mM PEP (Sigma‐Aldrich), 0.4 mM AMP (Sigma‐Aldrich), made up to a total volume of 200 μL in HEPES‐EDTA buffer at pH 7.0 (BioThema, Handen, Sweden). The reaction was started by adding the IPP/GPP substrate mixture. The increase in luminescence was measured continuously in relative light units (RLU) using a SpectraMax iD5 Multi‐Mode Microplate reader. Control experiments with different concentrations of tcFPPS, PPDK, GPP/IPP, and PEP were performed in order to confirm that all enzymes were active and the tcFPPS reaction was the rate limiting step under the conditions used.

The luminescent microplate assay was also used to measure the activity of the enzyme on an entire chip surface. The same immobilization procedure as described below for the SPR biosensor analysis was used, but manually pipetting the solutions onto the entire sensor surface with the chip outside the instrument (on the bench). Substrates (at concentration as above, but in SPR running buffer) were pipetted to the chip surface (see below). Samples of 10 μL each were taken every 10 s (10, 20 30, 40, and 50 s) and added to a standard reaction mixture in micro titer plate wells containing 0.32 U PPDK, 0.5 mM PEP, 0.4 mM AMP in HEPES‐EDTA buffer pH 7.0. Luciferin/luciferase from the assay kit (40 μL) was then added to each well, and the luminescence signal was measured in RLU using a SpectraMax iD5 Multi‐Mode Microplate reader.

### 4.7 *SPR biosensor analysis*

Analysis of direct interactions between tcFPPS and fragments was performed with a Biacore T200 instrument (GE Healthcare) at 25°C. tcFPPS was immobilized to a level of approximately 3,000 RU on CM5 Series S sensor chip using standard procedures for amine coupling.^33^ The running buffer used for immobilization, screening, and interaction analysis consisted of 10 mM HEPES, 150 mM NaCl, 3 mM Mg^2+^‐acetate, 1 mM TCEP, and 0.05% Tween20, supplemented with 1% DMSO for screening and interaction analysis. A reference screen was performed on a different surface from the screen, using the same buffer without Mg^2+^. A blank reference surface was activated and deactivated and used to detect non‐specific binding of fragments.

Fragments were diluted in running buffer and injected 250 μM for 60 s at a flow rate of 50 μL/min. The final DMSO concentration in all buffers used for screening was 1%. Nonspecific signals were removed by subtraction of sensorgrams from the reference channel. Compounds with signals between 30 and 100% of a theoretical *R*_max_ were selected as hits. The analysis was based on report points representing the average signal 5 s before the end of the injection (binding late [BL] response). The screening was performed with two different surfaces and 12 fragment hits were selected for further analysis. BL values were normalized with respect to the theoretical *R*_max_ of each fragment (as in Equation [1]), in order to account for differences in molecular weight and protein immobilization levels.^21^

(1) $$\left({\mathrm{RU}}_{\text{analyte}}\times \frac{\text{MWprotein}}{\mathrm{MW}\;\text{analyte}\times \text{Rprotein}}\right)\times 100\%.$$

The selected fragment hits were validated by analysis of a three‐fold dilution series starting at 250 μM for 60 s at a flow rate of 50 μL/min. Nonspecific signals were removed by subtraction of signals from the reference channel. Solvent corrections were also performed to compensate for differences in DMSO concentrations between the running buffer and samples. Apparent *K*_D_ values were estimated by a steady‐state analysis based on the BL response and using a standard equation, describing a simple 1:1 interaction model.

$$Y=\frac{B_{\max}\times X}{\left(K_{D}+X\right)}$$

A steady‐state analysis based on binding early response taken 7 s after the start of the injection was also performed, resulting in estimated *K*_*D*_‐values of the same order for each compound (Table 2).

Estimated *K*_*D*_‐values from report points were used to calculate the LE for each of the selected hits, according to Equation (2).

(2) $$\mathrm{LE}=\frac{\left(-\frac{\text{RTln}\left(K_{D}\right)}{n_{\mathrm{HA}}}\right)}{1000\left[\frac{\text{kcal}}{\mathrm{mol}}\times \mathrm{HA}\right]},$$

Where *R* is the gas constant 8.31451 J/mol·K, *T* is the temperature in Kelvin, and *n*_*HA*_ is the number of non‐hydrogen atoms in the sample molecules.^34^

The BE was calculated from steady‐state data at different ligand concentrations as the slope of the linear relationship between complex concentration and ligand concentrations at very low ligand concentrations.^21^ This results in an estimate of how much compound is bound as a function of concentration, without any assumptions of the mechanism of binding, number of binding sites or effects due to limited solubility of fragments at high concentrations.

## CONFLICT OF INTEREST

A.L. is employed by BioThema AB as CEO of the company that develops and sells firefly luciferase ATP reagents.
